# Editorial: Advances in protein structure, function, and design

**DOI:** 10.3389/fbioe.2022.1108962

**Published:** 2023-01-05

**Authors:** Ratul Chowdhury

**Affiliations:** ^1^ Department of Chemical Engineering, Iowa State University, Ames, IA, United States; ^2^ Nanovaccine Institute, Ames, IA, United States

**Keywords:** protein structure/folding, AlphaFold2, protein design and engineering, biological network, deep learning, AI-guided biology

Accessibility to ever improving computing infrastructure has led to a paradigm shift towards data-driven modeling in all areas of science and arts. Eponymously, data-driven modeling relies on 1) well curated, domain-knowledge-driven datasets, and 2) appropriate utilization of said data (*i.e.,* avoid overfitting, under sampling, *etc.*). The domain of protein biology has historically been on the lookout for a reliable method to discern the 3D-shape (structure) of a protein given its amino acid sequence. Precise knowledge of a protein’s structure enables us to first, explain how it works as a tiny molecular machine, and then devise rules to modify existing proteins or design new ones for therapeutic and engineering applications spanning–healthcare, green chemistry, energy, and novel functional materials.

One way to accurately determine the 3D-shape of a protein is *via* experiments (spectroscopy–Nuclear Magnetic Resonance, or crystallography–X-Ray diffraction) to catalog the 3D-Cartesian coordinates of each atom that are present in the protein. Such per-atom information is stored in a PDB (Protein Data Bank) format. Set up in 1976, the PDB (Berman et al., 2000) is a publicly accessible dataset of ∼198 k protein structures and has aggressively expanded at the rate of ∼11 k new entries per year since 2013. While this is quite a substantial dataset, this barely scratches the surface and constitutes only a meagre ∼.09% of the total set of 230M known protein sequences reported till date (UniParc dataset (Bairoch et al., 2005)). This has prompted the emergence of a gamut of data driven deep-learning techniques to reason over known sequence-structure pairs (from PDB) and create neural operators which can then predict the structure from any new protein sequence.

Two emerging, yet different schools of thought that fuel these deep-learning pipelines for structure prediction are: 1) family sequence alignment-based (FSA), and 2) single sequence-based (SS). FSA methods such as AlphaFold2 (Jumper et al., 2021) and RosettaFold (Baek et al., 2021) group all sequences a protein family (say, all amylases across species) and corresponding structures into constellations of similarity. During prediction, each input sequence is first sent through a sequence alignment pipeline to find which constellation it belongs to and use structures from the same constellation as templates to thread a possible predicted structure. Such methods, while powerful, cannot account for significant structural changes from point mutations unless such a mutant is a part of the training set (in which case it simply memorizes it). Interestingly, designed proteins with tailored function and disease-causing protein sequence variants fold into very different structures. Structural changes in these proteins are elusive to FSA structure predictors like AlphaFold2 and RosettaFold. On the other hand, SS methods (like RGN2 (Chowdhury et al., 2022)) use natural language processing to encode sequences to high-dimensional vectors and map such encodings to atomic coordinates of one (C α ) or more atoms (N, C, and C α ) of each amino acid. Since SS methods do not rely on similarity constellations, they are equipped to predict structural changes emerging from as little as a single amino acid change such as rapidly evolving viral proteins, and non-homologous *de novo* proteins. While neither FSA nor SS models are explicitly trained to be performative on structural changes arising from point mutations, at the limit of performance, SS models are better poised to capture such effects. Moreover, the biological event of protein folding upon synthesis is a molecular process which depends on the sequence of amino acids that make up the protein, not how similar proteins are folded in other species.

**FIGURE 1:**
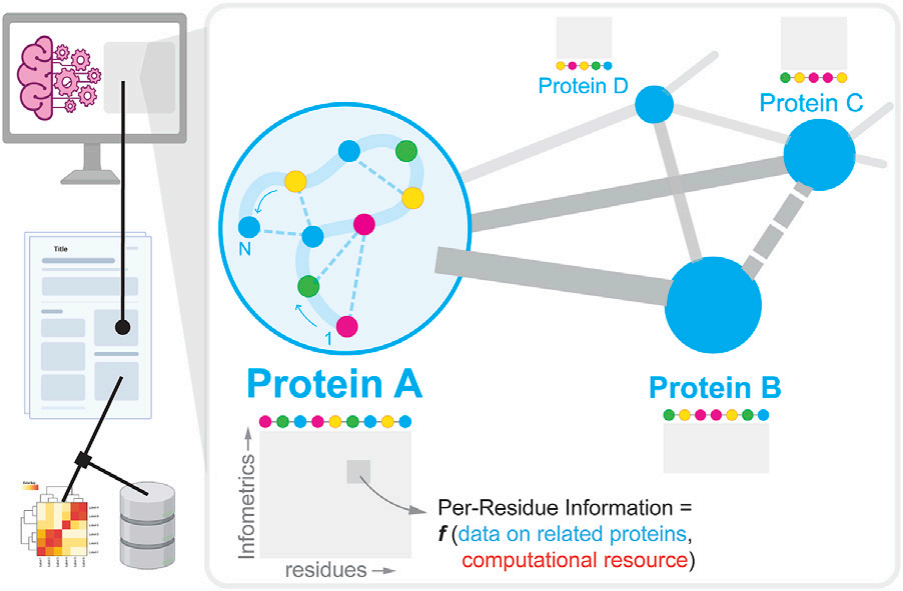
Overview of how experimental data describing a protein molecule and its interactions (inset) is reported in literature, and extracted using natural language processing to create datasets to support subsequent machine learning/AI-guided protein engineering efforts.

In this Research Topic, one of the key contributions is by Villalobos-Alva et al. which provides an extensive summary of ML/AI-based recipes and neural architectures that have been developed and deployed till date to learn, predict, and design proteins for various use cases (see Figure 1). Along the lines of structure prediction, Jin et al. show how Generative Adversarial Networks (GANs) can be utilized to predict secondary structure of proteins as a function of the inputs—1) amino acid sequence, and 2) similarity with known proteins with experimentally verified structure (i.e., structural prior). Next, Wang et al. first construe a protein structure as a network of amino acids either connected by covalent or non-covalent bonds and show how the network topology appended to node properties (of individual amino acids) can be mapped to protein function. The study reveals that DNA binding proteins tend to have buried hydrophobic pockets which are thermodynamically amenable to drug-binding. On the engineering front, Zhou et al. use information about active site of a particular enzyme (glutamate dehydrogenase) and tailors it to catalyze a stereoselective reaction with high efficiency. Pan et al. zoom out of a single protein and focuses on the entire protein-protein interaction (PPI) network in a single plant cell. In such a network, each node is a protein (and not the individual amino acids that make up the protein). A PPI dictates cellular phenotype *i.e.,* how an organism behaves as a consequence of proteins interacting with each other. They describe DWPPI–a deep walk algorithm that traverses a plant PPI to discern how information cascades through such a network.
